# Personalia

**Published:** 1914-09-05

**Authors:** 


**Personalia.**

Miss Luckes, the matron, has been presented with the badge of the British Red Cross Society, receiving at the same time a personal letter from Queen Alexandra.

Sir Edgar Vincent, until lately a member of the House Committee of the London Hospital, has been raised to the Peerage, under the title of Lord D'Abernon.

In order to express in some measure the appreciation of the House Committee of the London Hospital of the splendid services rendered by Lord Knutsford, the Chairman, they have decided to ask him to accept a painting of himself as a gift from them. Lord Knutsford has given sittings for this portrait, which will eventually hang in the Committee Room.

